# Dynamic patterns of communication and workload during cardiac surgery: An explorative study

**DOI:** 10.1371/journal.pone.0352703

**Published:** 2026-06-30

**Authors:** Liam Wietzorrek, Eugenie Craenen, Indy van Boven, Massimo A. Mariani, Fokie Cnossen

**Affiliations:** 1 Department of Cardiothoracic Surgery, Heart Centre, University of Groningen, University Medical Centre Groningen, Groningen, The Netherlands; 2 Department of Artificial Intelligence, Bernoulli Institute of Mathematics, Computer Science and Artificial Intelligence, University of Groningen, Groningen, The Netherlands; 3 Department of Anaesthesiology, University of Groningen, University Medical Centre Groningen, Groningen, The Netherlands; 4 Department of Cardiothoracic Surgery, Heart Centre, University of Utrecht, University Medical Centre Utrecht, Utrecht, The Netherlands; No institution, UNITED KINGDOM OF GREAT BRITAIN AND NORTHERN IRELAND

## Abstract

**Background:**

Effective communication amongst operating room (OR) teams is crucial for ensuring patient safety and optimizing surgical outcomes. This study aimed to investigate communication within the context of physiologically assessed workload during cardiac surgeries. Specifically, the relationship between team members’ workload and both case-relevant and case-irrelevant communication exchanges was explored, as well as their association with the length of surgical phases.

**Methods:**

Intraoperative communication exchanges were annotated from incision until skin closure across 24 recorded cardiac surgeries (>100h surgery time). OR team members wore heart rate monitors throughout the surgical procedures. HRV was calculated as the RMSSD across 5-minute non-overlapping windows. Mixed effect models were used to assess the relationship between workload and the frequency of communication exchanges across OR roles and surgical phases.

**Results:**

Relevant communication was most frequent during the initiation and discontinuation of extracorporeal bypass, while case-irrelevant communication was most frequent during the final closing phase of surgery. Team members were more likely to engage in case-irrelevant communication during periods of reduced workload (*χ*^*2*^ = 30.15, *p* = .001). However, this did not necessarily align with the workload of other team members, as increased workload was associated with more frequent case-irrelevant communication initiated by other team members (*χ*^*2*^ = 5.97, *p* = .015). Workload and case-relevant communication had a significant effect on surgical phase length.

**Conclusions:**

The findings highlight the dynamic relationship between workload and intraoperative communication patterns. Integrating observational data with physiological indicators of workload appears a promising approach to studying team dynamics in the operating room, though further research is needed to advance the use of such methods for improving team performance.

## Introduction

Non-technical skills, such as communication, teamwork and situational awareness, are crucial for patient safety and optimizing surgical outcomes [1,2]. Operating room (OR) teams during cardiac surgery consist of several interdependent sub-teams that must continuously communicate with each other to coordinate group-specific subtasks, while also collaborating on shared tasks. This distributed nature of tasks, along with limited visibility of others’ actions due to the OR layout and the need for individuals to maintain focus on their own responsibilities, makes effective communication essential for maintaining situational awareness and ensuring optimal team performance [3].

Communication in the OR can be distinguished by case-relevant communications (CRC) and case-irrelevant communication (CIC). CRC involves the exchange of information relevant to the current patient or surgical procedure, and allows for a shared understanding of the current situation and anticipated future states, crucial for patient safety [3,4]. On the other hand, case-irrelevant communication involves exchanges regarding work-related matters irrelevant to the current procedure, such as administrative tasks or other patients, as well as private conversations. CIC is one of the most frequent types of workflow disruptions in the OR [5] and can be distracting [6], hinder effective teamwork [7] and have a negative effect on the surgical outcome [4].

Excessive workload and stress can impede both technical and non-technical skills in the OR [8], where, for example, more surgical errors [9], worse decision making [10] and poorer situational awareness can be observed under high workload conditions [10]. According to the cognitive load theory, it is when the cognitive load, determined by the task, the individual and their environment, outweighs the finite cognitive capacities available, that task performance suffers and the chance of errors can increase [11]. When CIC becomes a distraction in the OR, it can increase the extraneous load placed upon individuals. For example, research on robot-assisted surgery found that anaesthesiologists reported greater stress due to CIC [12]. On the other hand, CIC is also suggested to be a coping mechanism that takes place during periods of low workload, as more CIC has been observed during surgical cases that were perceived as less demanding [5–7].

However, research addressing the relationship between communication and intraoperative workload have often relied on self-report methods, which may be less sensitive to fluctuations in workload over time and more susceptible to self-report biases [13]. Therefore, physiological assessments of workload appear to be a promising approach for studying team dynamics in the OR, as they allow for continuous real-time measurements [13–15]. There remains debate regarding the specificity of physiological measures to solely capture mental workload, as various other factors are also likely to affect the physiological measurements, such as physical exertion, emotional arousal and environmental factors [16]. However, numerous studies have successfully applied heart-rate variability (HRV) as a measure of stress and cognitive workload in the OR across various surgical specialities, including cardiac surgery [13,15,17–20]. HRV measures the variation of consecutive inter-beat intervals, driven by the dynamic changes of the sympathetic (SNS) and parasympathetic nervous systems (PNS) [21]. A commonly used metric of HRV that strongly correlates with PNS activity is the Root Mean Square of Successive Differences (RMSSD), where an increase in RMSSD reflects an increase in PNS activity indicative of reduced stress [22].

While self-report methods have been used to study non-technical skills and workload in the OR, there is a lack of research applying physiologically assessed workload to assess intraoperative team dynamics [8,13,23]. To address this gap, this study explores patterns of communication and workload by incorporating observational and HRV data from cardiac surgery OR teams. In line with a safety II approach to safety management, rather than focusing on errors and normative prescriptions of behaviour, this study will explore team dynamics by observing intraoperative behaviours as is actually performed during real-life surgery [24]. Specifically, we aim: (1) to investigate differences in communication and workload across distinct surgical phases and OR team roles; (2) to explore the relationship between workload and intraoperative communication at a finer temporal resolution by utilising HRV measurements as a continuous indicator of workload, where exchanges will be distinguished according to case relevance and team member involvement; and (3) to assess whether workload and the frequency of CRC and CIC are associated with the length of surgical procedures, as longer surgical procedures can have implications for both patient safety and health care delivery, particularly in the context of limited resources and patient turnover rates [25]. Understanding how workload and communication interact during surgery has direct implications for patient safety and surgical outcomes. By identifying patterns of workload and communication across surgical phases and roles, and exploring their relationship at a fine-grained level, this study aims to provide insights that can inform strategies to improve team performance and reduce errors. Table 1 provides an overview of the abbreviations and definitions used in the paper.

**Table 1:** A list of abbreviations and acronyms used in the paper.

Abbreviation	Definition
CIC	Case-Irrelevant Communication
CPB	Cardio-pulmonary bypass
CRC	Case-Relevant Communication
ECG	Electrocardiogram
HRV	Heart rate variability
LMM	Linear mixed model
OR	Operating room
RMSSD	Root mean square of successive differences
RR	R-R interval, i.e., time between two consecutive R peaks in ECG signal

## Materials and methods

### Participants

Patients and OR staff provided written informed consent in accordance with the Central Ethics Review Committee of the University Medical Centre Groningen (CTc UMCG, ID: 202100238). Recordings only took place with prior consent from the patients and operating room staff, between the 1^st^ of December 2022 and the 31^st^ of February 2024.

Naturalistic observations were conducted with audio-visual recordings for a total of 29 cardiac surgeries. 24 were included in the final dataset and used for subsequent analysis. Exclusion criteria include: No heart rate recording of the lead surgeon, due to malfunctioning heart rate sensors or withdrawn consent prior to incision (*N* = 2), surgical cases without extracorporeal circulation, as the procedure differs substantially from cases involving extracorporeal circulation (e.g., absence of perfusionist) (*N* = 2), incomplete recordings due to technical problems, where the surgical data recording system partly malfunctioned (*N* = 1). Included surgical cases include Bypass surgeries (*N* = 9), Valve Repair Surgeries (*N* = 6), Bypass and Valve Repair surgeries (*N* = 4), Aortic Root Replacements (Lansac procedure) (*N* = 3), LVAD surgery (*N* = 1), and myxoma excision surgery (*N* = 1). The final dataset includes the analysis of 101.6 hours of recorded surgery time. Individual recordings ranged from 3.07h to 5.42h (*M* = 4.23h, *SD* = 0.85h)

Across the 24 surgeries recorded, core OR team members comprised 72 different individuals. Depending on the presence of undergraduate students, observing residents, and trainees, the number of individuals present in the OR ranged from 7 to 14.

Except for circulating nurses, all core OR team members were asked to wear a Polar H10 chest strap to record their ECG data. Although circulating nurses are an integral component of the operating room team, they were excluded from HRV recordings for logistical reasons, including frequent staff rotations and the large number of individuals involved. Consent for ECG recordings was collected separately from the consent for audio-visual OR recordings. ECG data was collected from a total of 53 OR team members. This study utilises the ECG data from the anaesthesiologists, perfusionist and surgeons, as representatives of the three main sub-groups during cardiac surgery, which includes six anaesthesiologists, seven perfusionists and eleven surgeons. All 24 surgical cases include ECG data from the surgeon and perfusionist. Six cases do not include ECG data from the anaesthesiologist, due to technical difficulties, where the Polar devices did not adequately measure participants ECG, or a lack of consent, where anaesthesiologists consented to being recorded but did not consent to wearing the ECG recorders. By ensuring complete confidentiality, we aimed to protect the safe working culture in the OR and reduce the likelihood that participants would change their behaviours due to the presence of the recording devices.

### Materials

**Fig 1:**
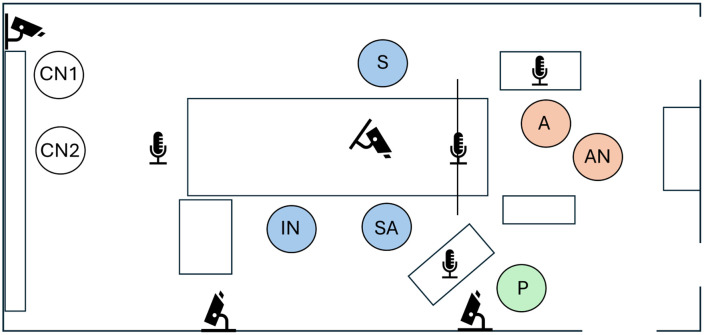
OR layout showing camera & microphone placements and a typical core OR team. S = surgeon; SA = surgical assistant; IN= Instrument Nurse; A = Anaesthesiologist; AN = Anaesthesia Nurse; CN = Circulating Nurse. The patient table is centred in the operating room, with the surgical team (blue) around it, the anaesthesia team (orange) at the patient’s head behind the sterile drape, and the perfusionist (green) operating the heart-lung machine.

Four cameras with built-in microphones were installed in the operating theatre, with one camera directed at the incision site and three wide-angle cameras covering the entire operating room. Additionally, four voice recorders (Olympus VN711PC + Olympus ME-52/ RØDE VMPR) were placed at relevant locations in the operating room to improve the recording quality of verbal communications (see Fig 1), namely at the foot of the patient, the heart-lung machine, the sterile drape and the anaesthesia cart.

ECG data from the polar H10 chest straps were continuously sent via Bluetooth to the “Polar Sensor Logger” application [26] on an android smartphone. Kubios software [27] was used to calculate HRV metrics from the ECG data. R was used for the statistical analysis [28], including the LMER, glmmTMB, CAR, and emmeans packages.

### Analysis

ECG, audio and video recordings were synchronized and analysed from initial skin incision until final skin closure. Kubios software [27] was used to calculate HRV metrics from the RR intervals (i.e., time intervals between consecutive R peaks in the ECG signal). Noise detection and artefact corrections were also performed by automatic correction algorithms in Kubios. Kubios was chosen because it is a widely validated and commonly used software package, offering robust preprocessing and HRV analysis methods consistent with current guidelines [27,29,30]. The Root Mean Square of Successive Difference (RMSSD) was used as the psychophysiological measure of workload, as the RMSSD is predominantly driven by parasympathetic activity and has been used to assess workload in similar real-world settings with promising results [19,31,32]. RMSSD values were calculated for 5-minute non-overlapping windows, consistent with standard short-term time-domain HRV methodology to ensure stable estimation of variability indices while maintaining adequate temporal resolution [33]. To account for individual differences, workload was normalized with respect to the individual’s range of RMSSD values across the entire surgery (i.e., Min-Max Scaler). As greater RMSSD indicates lower stress, normalized RMSSD values were inverted, with values nearing 1 indicating an individual’s maximum workload and values nearing 0 indicating minimum workload during the surgery [31].

Audible communication in the operating room was transcribed and relevant events annotated, such as procedural milestones and the task-related context of communication exchanges. All collected data was anonymized. Communication exchanges were split into categories of relevant communication and case-irrelevant communication. Case-relevant communication (CRC) was defined as all communications pertaining to the current procedure, while case-irrelevant communication (CIC) included all other communications not relevant to the current procedure, including both small-talk and work-related issues not directly pertaining to the current surgical case.

To assess the relationship between workload and intraoperative communication, the frequency of communication exchanges was assessed at the 5-minute level, corresponding with the 5-minute HRV segments. Exchanges were further distinguished by “engaging in communication” and “others’ communication”. “Engaging in communication” was defined as the number of communications in which the anaesthesiologist, perfusionist or surgeon actively participated in the verbal exchange. To examine whether the workload is affected by “others’ communication”, the frequency of communications in the OR was assessed in which the anaesthesiologist, perfusionist or surgeon did not actively participate in the interaction exchange. This distinction was made to assess active engagement in communication, which may reflect cognitive involvement and coordination demands, whereas others’ communication may represent background interaction that could contribute to environmental complexity and noise. Differentiating these categories allows for a more nuanced analysis of how communication dynamics relate to workload. Communications were coded by two researchers, with backgrounds in human factors and communication science, using a double-coding approach. After jointly coding an initial subset to calibrate the framework, both coders independently analysed the dataset. Any disagreements were addressed iteratively through open discussions until a consensus was reached. Therefore, the final annotations represent a shared interpretation of the data.

Furthermore, five surgical phases were defined, as: 1. Sternotomy, from skin incision until initiation of cannulation; 2. cannulation/initiation of CPB, from cannulation until aortic clamping; 3. Intracardiac repair, from aorta clamp placement until removal; 4. decannulation/termination of CPB, from aorta clamp removal until decannulation; 5. Closure, from decannulation until skin closure. These surgical phases were selected because they represent procedural milestones for the OR team and are universally relevant to surgeries involving cardiopulmonary bypass.

The effects of workload and communication on surgical phase length were assessed at the “phase-level”, where communication was defined as the mean number of all CRCs or CICs observed in the room per minute for each surgical phase. Workload at the “phase-level” was calculated as both the mean of all 5-minute segments within each surgical phase (i.e., “mean workload”,) and the maximum value amongst all 5-minutes within each surgical phase (i.e., “maximum workload). The mean and maximum across observations within a phase provide summary measures intended to capture the overall level of communication frequency and workload, which could then be related to the corresponding duration of surgical phases.

### Statistics

Mixed effect models were used to explore communication and workload across OR roles and surgical phases. All models included a random effect term for the surgical cases to account for the repeated measures design.

Differences in workload across surgical phases and OR roles were examined with linear mixed effect models (LMM) for both the mean and maximum workload measured within each surgical phase. The frequency of all CRC and CIC in the OR was examined across surgical phases with negative binomial Poisson models. An offset term for the varying length of surgical phases was included.

The association between workload and communication frequency was assessed at the 5-minute level with zero-inflated Poisson models to account for the inflated number of 5-minute segments that contained no communication exchanges of interest. Negative Binomial models were used to account for over-dispersed CRC data. Workload, surgical phase and OR role, as well as their interactions, were modelled as fixed effects, while surgical case and phase were modelled as nested random effects.

LMMs were used to assess the effect of communication frequency and workload on phase length. For the effect of communication on phase length, the mean frequency of CRC and CIC in the OR across surgical phases was considered. The effect of workload on phase length was modelled with the mean and maximum workload across surgical phases and OR roles. Model assumptions were assessed with residual plots and phase length was log-transformed to normalise the data.

All models were fit using the LMER or glmmTMB packages in R [28]. Omnibus Wald Chi-square tests were conducted with the CAR package, to summarise model results across the factor levels. Post-hoc analyses were conducted with the emmeans package. All tests were conducted at α = 0.05 and p-values were adjusted with the Sidak method for multiple comparisons.

## Results

In the first part of this section, we investigate whether different surgical phases were associated with different levels of workload for different team members, and whether different surgical phases were associated with differences in case-relevant and case-irrelevant communication, before describing their interactions. In the second part, we describe whether workload and communication affect the length of surgical phases.

### Cognitive workload across phases

**Fig 2:**
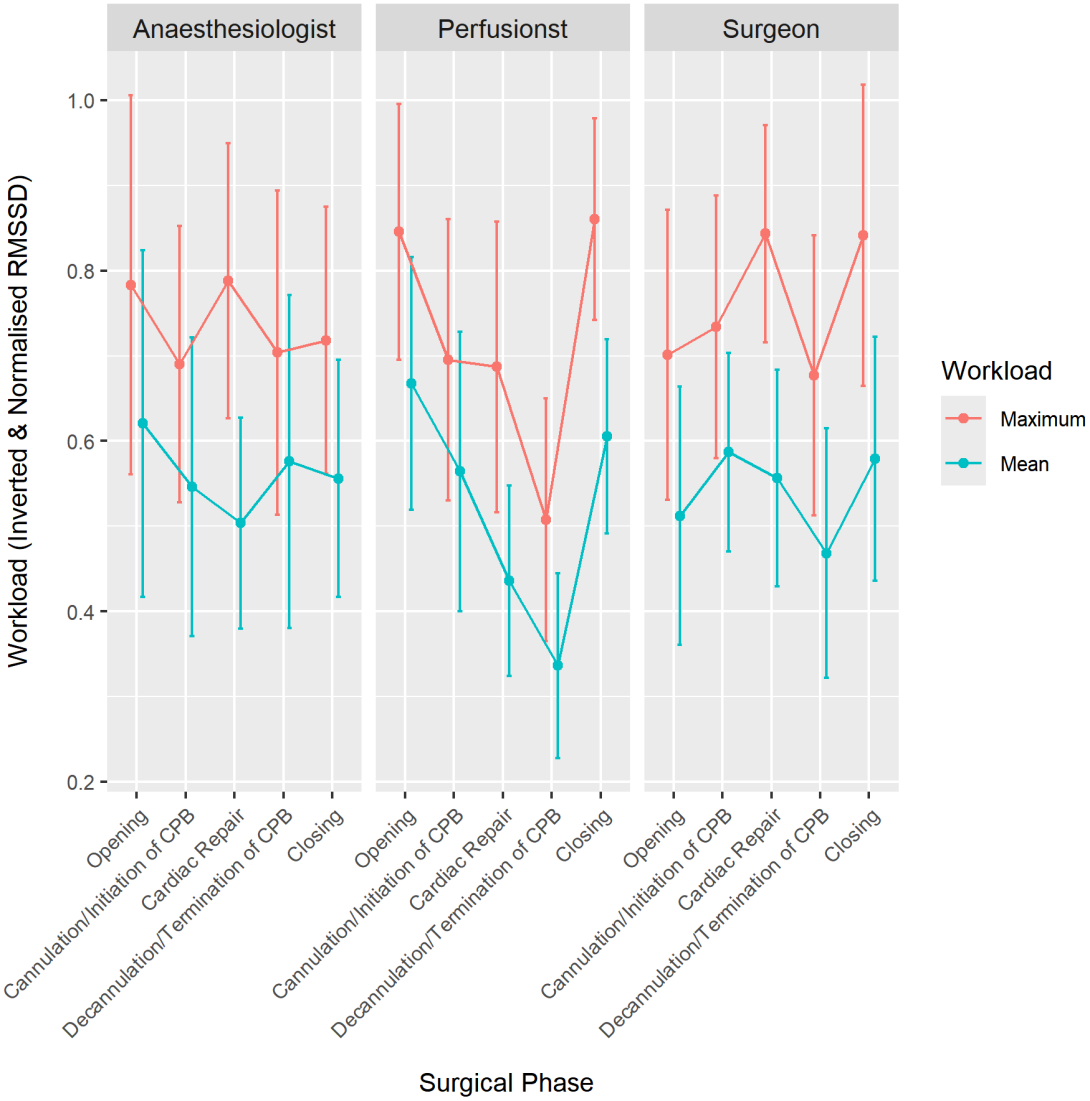
Mean and maximum workload by surgical phase and OR role. + /- 1SD.

Different team members experience different workload levels in different phases of the procedure: the interaction between surgical phase and role had a significant effect on the mean workload (𝜒^2^(8) = 51.84, *p* < .001) and maximum workload (𝜒^2^(8) = 48.97, p < .001) (see Fig 2). Post-hoc comparisons showed that surgeons experienced significantly lower mean workload (β = −0.156, *p* < .003) and maximum workload (β = −0.146, *p* = .025) than perfusionists during the sternotomy phase. During the decannulation/termination of CPB phase, perfusionist experienced significantly lower workload than anaesthesiologists (Max. Workload: β = −0.199, *p* = .001; Mean Workload: β = 0.242, *p* < .001) and surgeons (Max. Workload: β = −0.169, *p* = 004; Mean Workload: β = 0.132, *p* = .021). Finally, surgeons experienced significantly greater maximum workload than perfusionists during the intracardiac repair phase (β = 0.156, *p* = .011).

### Communication across phases

**Fig 3:**
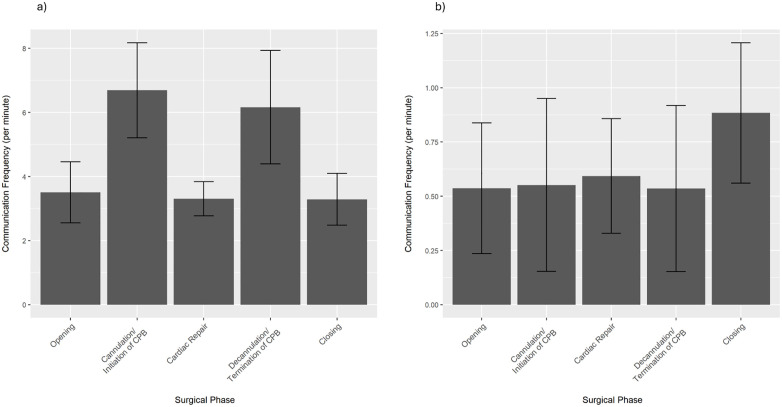
(A) Mean frequency of case-relevant communication per minute across surgical phases; (B) Mean frequency of case-irrelevant communication per minute across surgical phases. + /- 1 SD.

Averaged across team members, *case-relevant communication* in the OR showed significant differences across surgical phases (𝜒^2^(4) = 368.919, *p* < .001). Relative to other surgical phases, CRC was most frequent during the cannulation/initiation of CPB and the decannulation/termination of CPB (Fig 3A). Surgical phase also had a significant effect on *case-irrelevant communication* (𝜒^2^(4) = 31.431, *p* < .001), where CIC was most frequently observed during the closing phase of surgery (Fig 3B).

**Fig 4:**
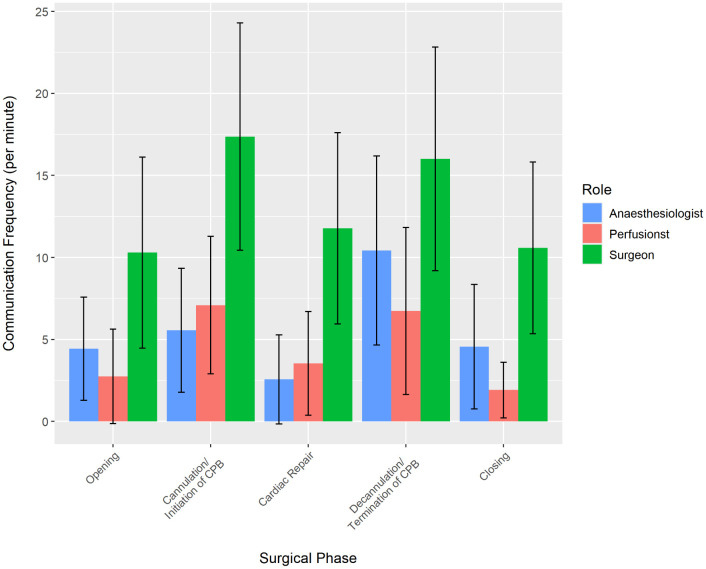
Mean frequency of relevant communication by OR role and surgical phase. + /- 1 SD.

Fig 4 shows the frequency of *case-relevant communication* for the anaesthesiologist, surgeon and perfusionist separately across surgical phases. Overall, surgeons communicated most frequently. Communications exchanges involving the surgeon were most frequent during the cannulation/initiation of CPB and the decannulation/termination of CPB. This was also the case for perfusionists. Anaesthesiologists communicated most frequently during decannulation/termination of CPB and least frequently during the intracardiac repair phase.

### Communication & cognitive workload across phases

We next investigated whether the associations between workload and communication were different across surgical phases and for different OR team members, for case-relevant and case-irrelevant communication separately. We also analysed this when communication was exchanged by others than the OR team member.

#### Workload and engaging in communication.

**Fig 5:**
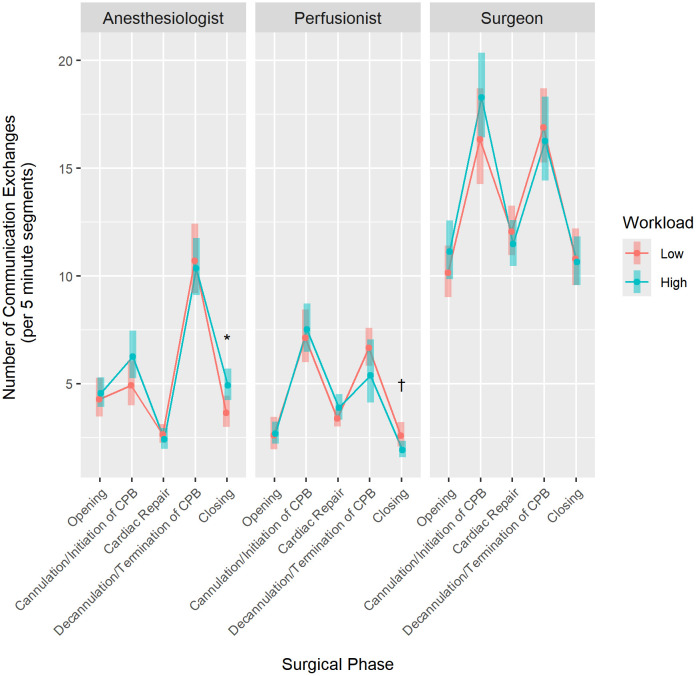
Estimated marginal means for the predicted frequency of engaging in case-relevant communication per 5-minute segment across workload, surgical phase and OR role. Low and high workload represent the 25th and 75th percentile of workload across 5-minute segments. † = p < 0.1; * = p < 0.05; ** = p < 0.01; *** = p < 0.001; + /- 1SE.

The three-way interaction of OR role, surgical phase and workload showed a significant effect on the frequency of *case-relevant communication* (CRC) (Table 2a). Post-hoc analyses showed that, when averaged across OR roles, more CRC was significantly associated with increased workload during the cannulation/initiation of CPB phase (β = .443, *p =* .033). Considering simple slopes at each level of phase and role, anaesthesiologists engaged more frequently in CRC with increased workload during the closing phase of surgery (β = 0.965, *p* = .028). Workload had no significant association with related communication at all other combinations of OR role and surgical phase (Fig 5).

**Table 2:** Omnibus Wald Chi-square test for the effect of workload on engaging in (a) case-relevant and (b) case-irrelevant communication across OR roles and surgical phases.

*(a) CRC*	*Chisq.*	*Df*	*p*	*(b) CIC*	*Chisq.*	*Df*	*p*
(Intercept)	1154.82	1	<.001	(Intercept)	3.143	1	.076
**workload**	**0.764**	**1**	**.382**	**workload**	**30.151**	**1**	**<.001**
role	212.247	2	<.001	role	23.428	2	<.001
phase	111.245	4	<.001	phase	8.042	4	.090
**workload:role**	**6.270**	**2**	**.044**	**workload:role**	**0.306**	**2**	**.858**
**workload:phase**	**12.941**	**4**	**.012**	**workload:phase**	**10.522**	**4**	**.032**
role:phase	40.563	8	<.001	role:phase	17.028	8	.030
**workload:role:phase**	**25.524**	**8**	**.001**	**workload:role:phase**	**8.226**	**8**	**.412**

Note: Boldface indicates main effects and interaction effects of workload.

**Fig 6:**
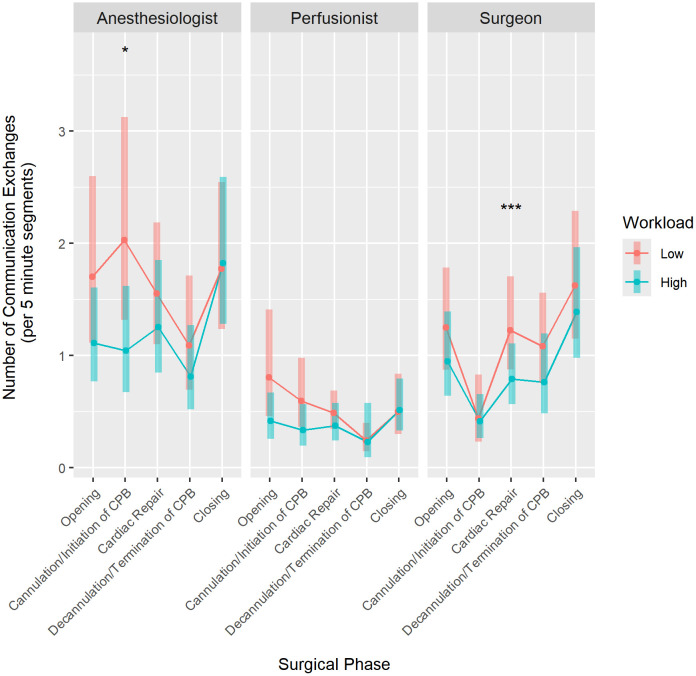
Estimated marginal means for the predicted frequency of engaging in case-irrelevant communication per 5-minute segment across workload, surgical phase and OR role. Low and high workload represent the 25th and 75th percentile of workload across 5-minute segments. † = p < 0.1; * = p < 0.05; ** = p < 0.01; *** = p < 0.001; + /- 1SE.

For *case-irrelevant communication*, workload showed a significant main effect, as well as a significant interaction effect with surgical phase (Table 2b). All three OR roles reduced their engagement in CIC during greater workload, averaged across surgical phases (Anaesthesiologists: β = −1.022, *p* < .001; Perfusionists: β = −0.995, *p* = .024; Surgeons: β = −0.831, *p* = .003). Considering simple slopes at each level of phase and role, significant negative associations were present for anaesthesiologists during the cannulation/initiation of CPB (β = −2.163, *p* < .001), and for surgeons during the cardiac repair (β = −1.418, *p* < .001) (Fig 6).

#### Workload and others’ communication.

**Fig 7:**
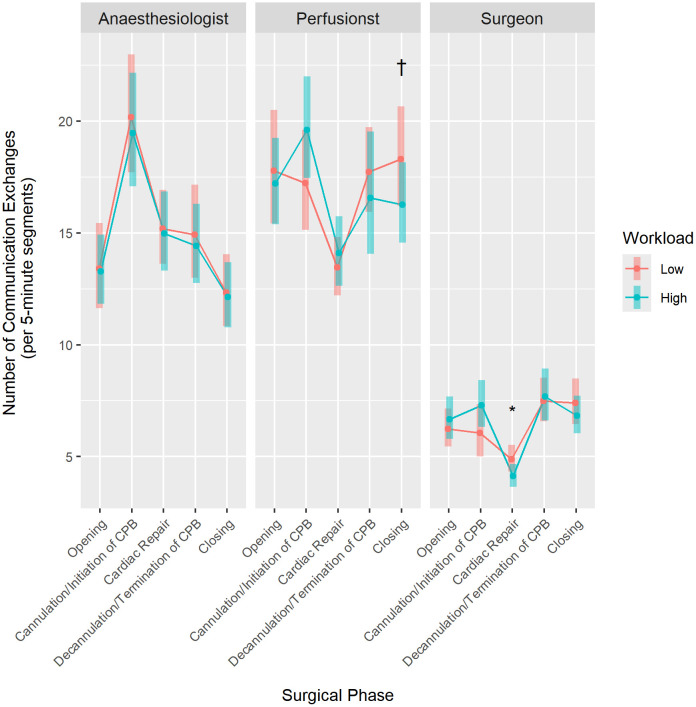
Estimated marginal means for the predicted frequency of others’ case-relevant communication per 5-minute segment across workload, surgical phase and OR role. Low and high workload represent the 25th and 75th percentile of workload across 5-minute segments. † = p < 0.1; * = p < 0.05; ** = p < 0.01; *** = p < 0.001; + /- 1SE.

Workload showed a significant interaction effect with surgical phase and OR role on the frequency of *case-relevant communication* by other team members (Table 3a). Post-hoc analyses showed less CRC by others was associated with greater workload for surgeons during the cardiac repair phase (β = −0.555, *p* = .012)(Fig 7). All other combinations of OR role and phase showed no significant association between workload and others’ case-relevant communication.

**Table 3:** Omnibus Wald Chi-square test for the effect of workload on others (a) case-relevant and (b) case-irrelevant communication across OR roles and surgical phases.

(a) CRC	*Chisq*	*Df*	*P*	(b) CIC	*Chisq*	*Df*	*p*
(Intercept)	2791.013	1	<.001	(Intercept)	13.741	1	<.001
**workload**	**0.309**	**1**	**. 578**	**workload**	**5.971**	**1**	**.015**
phase	9.152	4	. 057	phase	17.194	4	.001
role	209.046	2	<.001	role	15.210	2	<.001
**workload:phase**	**12.670**	**4**	**. 013**	**workload:phase**	**5.915**	**4**	**.206**
**workload:role**	**0.497**	**2**	**.780**	**workload:role**	**3.094**	**2**	**.213**
phase: role	36.485	8	<.001	phase:role	14.742	8	.064
**workload:phase:role**	**22.305**	**8**	**.004**	**workload:phase:role**	**12.445**	**8**	**.132**

Note: Boldface indicates main effects and interaction effects of workload.

**Fig 8:**
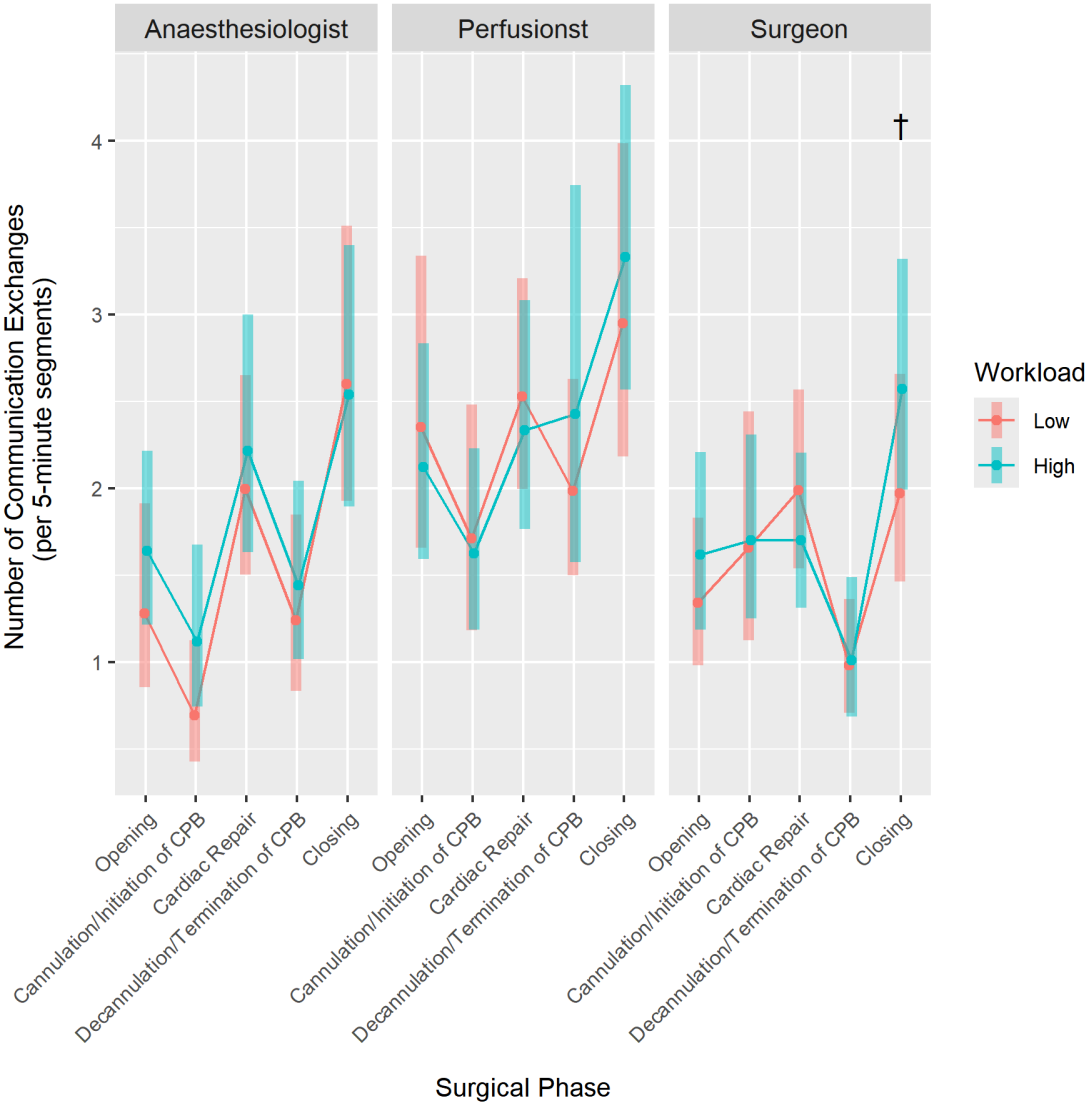
Estimated marginal means for the predicted frequency of others’ case-irrelevant communication per 5-minute segment across workload, surgical phase and OR role. Low and high workload represent the 25th and 75th percentile of workload across 5-minute segments. † = p < 0.1; * = p < 0.05; ** = p < 0.01; *** = p < 0.001; + /- 1SE.

Workload showed a significant main effect on Other’s *case-irrelevant communication*, where more CIC by others was associated with greater workload (β = .303, *p* = .015) (Table 3b). After p-value correction, post-hoc analyses across surgical phase and OR role did not reach statistical significance (Fig 8).

### Length of surgical phases

To assess the effect of communication frequency and workload on phase length, we used linear mixed effect models.

The frequency of all *case-relevant communication* in the OR showed a significant main effect on phase length, where more frequent CRC was associated with shorter surgical phases (β = −0.13, p = .006). More frequent CRC was associated with a shorter decannulation/termination of CPB phase (β = −0.202, p < .001). The frequency of *case-irrelevant communication* in the OR showed no significant association with the length of surgical phases.

The mean *workload* per surgical phase experienced by the surgeon, anaesthesiologist and perfusionist showed no significant effect on surgical phase length. The maximum workload per surgical phase by OR role showed a significant main effect on phase length, where greater maximum workload was associated with longer surgical phases (β = 0.674, p < .001). For surgeons and anaesthesiologists, greater maximum workload was associated with a longer cannulation/initiation of CPB phase (Anaesthesiologist: β = 1.250, *p* = .008; Surgeon: β = 1.252, *p* = .005) and decannulation/termination of CPB phase (Anaesthesiologist: β = 1.053, *p* = .022; Surgeon: β = 1.055, *p* = .021).

## Discussion

This study set out to explore the relationship between intraoperative workload and case-relevant and irrelevant communication, as well as whether these factors influence the length surgical phases. The results show distinct patterns in both workload and communication across different surgical phases and OR roles.

Across all phases, the surgeon was most frequently involved in communication exchanges. This coincides with previous research that found the surgical group to be the focal point of communication in the OR [34]. Furthermore, as CRC was most frequent during the cannulation/initiation and decannulation/discontinuation of CPB phases, our results emphasize the importance of communication during these phases due to the interdependent nature of the tasks performed.

Case-irrelevant communication was most frequent during the final phase of surgery. Possible reasons include a more relaxed social climate, reduced concentration demands, a sense of accomplishment, or increased fatigue as the surgery nears its end [35,36]. Case-irrelevant work-related matters, such as a lack of clarity over the OR schedule or preparations for the following patient, may have also become more pertinent during the final stages of surgery, which could further contribute to our findings.

The differences in HRV-assessed workload indicate contrasting task demands placed upon each team member at different stages of surgery. Interestingly, the surgeon and perfusionist experienced relatively low workload during the decannulation/termination of CPB phase, which contrasts with findings from self-report research [37]. Reduced workload was observed when surgeons experienced a moment of recovery after completing the demanding cardiac repair, while waiting for patient hemodynamics to stabilize and the echocardiographic assessment of adequate heart functioning. Furthermore, physical exertion may also influence the workload assessment, as perfusionists showed their greatest workload while assembling and disassembling the CPB pump during the initial and final phases of surgery.

Differences between the maximum and mean workload became particularly evident during the cardiac repair phase. When assessing maximum (inverted) HRV, anaesthesiologists and surgeons experienced the greatest workload during the cardiac repair phase, which corresponds with previous research investigating HRV-assessed peaks in workload during cardiac surgery [19]. Averaging workload can obscure periods of elevated workload due to moments of relaxation occurring within the same phase, particularly during longer phases. Therefore, our assessments at a finer temporal resolution, i.e., 5-minute intervals, may have allowed for more nuanced insights into workload and its relationship with team dynamics.

The results show that team members are more likely to engage in case-irrelevant talk when they are experiencing low workload. Self-report studies have shown similar findings [5–7,35]. Engaging in CIC may serve as a strategy to stay active during moments of low workload, where team members might use these opportune moments to relieve tension, maintain interpersonal relationships or divert attention to tasks unrelated to the current surgical case [5,6,38].

However, given the potentially conflicting periods of high and low workload across different team members, others’ conversations could also become distracting to individual team members [6,12,39,40]. While case-relevant communications may be necessary and unavoidable, team members would ideally recognise the increased workload of others and reduce their case-irrelevant conversations. However, we were not able to observe this effect in the data. Instead, our results showed a significant main effect suggesting that team members experienced greater workload as other’s CIC increased, which was not observed for others’ CRC. Echoing findings from previous research, team members may not always recognise the workload of others or express their need for silence in the OR [5]. Hence, while the initiation and discontinuation of CPB have been acknowledged as critical “sterile cockpit” phases of surgery [37], a more nuanced approach, such as improving awareness of fluctuating workload demands within the team, could also help prevent extraneous factors from becoming disruptive [5,35].

The relationship between case-relevant communication and workload appeared more variable, where the surgical phases and their defining task demands likely play an important role. For example, critical shared tasks, such as the cannulation and initiation of CPB, can elicit greater workload while also requiring effective communication and coordination amongst the team. On the other hand, demanding solitary tasks can result in increased workload, while communication in the OR naturally decreases. Examples include the surgeon performing the anastomoses during the cardiac repair phase or the perfusionist disassembling the CPB machine during the closing phase.

Finally, while the mean workload per phase showed no association with phase length, a greater maximum workload was associated with longer phase length of the cannulation/initiation of CPB, decannulation/termination of CPB. Hence, greater maximum workload may more adequately capture increased task demands that result in longer phase length [41]. CIC had no effect on surgical phase length. This could reflect the OR team’s ability to compensate for CIC in the room by maintaining focus and blocking out noise in the room, which prevents CIC from hindering case progression [42]. On the other hand, more frequent CRC was associated with shorter phase length, which may reflect more efficient team processes, as more frequent information exchanges can help create a shared understanding and contribute towards more cohesive teams [4,41]. However, other factors, such as case complexity, are likely to influence both surgical phase duration and communication frequency, highlighting the need for further research to explore their relationship.

Taken together, this study highlights the potential of assessing intraoperative team behaviours within the context of workload. Such research can enhance the awareness of OR teams towards their patterns of workload and communication throughout the procedure, enabling teams to learn and adapt more effectively. For example, training programs, guided by physiological measurements, and focusing on mutual awareness, shared mental models and the recognition of (non-verbal) cues of high workload of other team members, as well as how to adapt accordingly, may benefit team performance and patient safety in OR. Similarly, the adoption of standardised communication protocols in analogy to “mayday” and “panpan” from the aviation industry may help provide a safe manner to inform the team of high workload situations and trigger appropriate responses [43,44]. Importantly, surgical data recorders offer novel opportunities for performance feedback that can be used for the training of OR teams and residents, such as structured video-assisted debriefing sessions [45], while the integration of physiological workload can provide critical insights into the demands placed on operating room (OR) teams. For example, surgical education may benefit from continuous physiological assessments to optimize training platforms by monitoring trainees’ cognitive load and dynamically adjusting training parameters in real time, which can enhance skill acquisition and retention [46]. Furthermore, surgical data recorders enable a data-driven approach to understanding team dynamics and performance in the operating room. Such data can be used for real-time feedback during surgery and presented as interactive dashboards for OR teams, representing early steps toward the implementation of cognitive aid systems [47]. These systems could include adaptive workload displays and intelligent interruption management tools that leverage cognitive load measurements to mitigate the negative effects of interruptions.

Finally, it should be noted that while this study focused on cardiac surgery, the underlying dynamics between workload and communication may not be unique to this domain. The data driven methodology employed in this study, including the integration of physiological workload measures with recordings of team behaviour, appears a promising approach in various other high-stake team environments, including maritime, aviation, emergency response and broader healthcare settings [48–51]. Our results focus on cardiac surgery, where the distinct nature of the surgical task and the multidisciplinary team composition shape communication and workload patterns. Nonetheless, certain observed team dynamics, such as the importance of maintaining awareness of others’ workload and the occurrence of case-irrelevant communication during low workload, may reflect domain-general principles related to the coping mechanisms and adaptability of resilient high reliability teams [52,53]. These principles become particularly relevant in high-stake environments where safe communication practices and workload management strategies are crucial for safety and team performance. This highlights opportunities for interventions within the wider human factors field that leverage continuous workload measurements to study and train team performance, enabling dynamic cognitive load management and evidence-based strategies to optimize coordination in complex environments.

### Limitations

Due to the observational nature of the study, it is not possible to rule out the possibility that participants modified their behaviours due to the presence of the recording devices. Such a Hawthorne effect could have led to more socially desirable behaviours during recordings, for example, reducing case-irrelevant communication. Furthermore, results may not be generalisable to other settings due to the data sample being collected at a single medical centre. Surgical cases differed by type of procedure, team size and team composition, which is likely to affect both cognitive workload and communication and account for a large portion of variation in the data. Unexpected events or complications at different stages of surgery also introduce variability in the data, as do individual differences within the same OR role, such as experience and coping mechanisms. Furthermore, as this study was exploratory in nature, the number of post-hoc comparisons may have resulted in reduced statistical power and limited our ability to adequately detect significant differences in the data. Future research could build upon these initial exploratory findings by collecting larger datasets, from multiple medical institutions, to improve the generalizability of the findings and allow for a more comprehensive study of communication and workload by including additional contextual variables, such as various surgical specialties, teaching scenarios, emergency surgeries and further team interaction variables

Another limitation concerns the focus on communication frequency, without exploring the quality of communication. Future research could address safety-relevant features of CRC, such as read-back and non-ambiguous communication [34], in the context of HRV-assessed intraoperative workload. Similarly, case-irrelevant communication could be differentiated into work-related and social CIC [35], which could help highlight the effect of other work-related tasks on intraoperative workload. In addition, non-verbal communication, such as gestures and eye contact, plays a critical role in team coordination in the operating room but was not captured in this study. Future research should aim to include these non-verbal elements to provide a more comprehensive understanding of communication dynamics.

Another important limitation of this study is the inability to definitively attribute changes in HRV to cognitive demands alone. HRV is likely influenced by multiple factors present in the OR, including emotional stress, physical exertion and environmental conditions. Therefore, it is possible that HRV variations in this study reflect a composite of task-related strain and cognitive workload. For example, perfusionists elevated workload while assembling the CPB pump could reflect both cognitive processes as well as physical exertion. These various influences on HRV should be considered when interpreting the findings, as it may affect the precision of HRV as a measure of solely cognitive workload. Future research should incorporate complementary measure to better disentangle these contributing factors.

Finally, it is important to note that this study employed a naturalistic observational methodology with correlational analyses. As such, definitive conclusions regarding causality cannot be drawn. Future research could employ experimental designs or structural equation modelling to further investigate the dynamic relationship between workload and non-technical skills.

## Conclusion

This study explored communication and workload during cardiac surgeries by integrating audiovisual recordings and HRV measurements of OR teams. Workload and communication patterns differed across surgical phases and OR roles. Results also highlighted the dynamic relationship between intraoperative communication and workload. Notably, while individual workload influenced the occurrence of case-irrelevant communication, this did not always align with the workload experienced by others, potentially becoming a source of disruption during periods of high workload. This methodological approach demonstrates the value of capturing real-time behavioural and physiological data to assess non-technical skills during routine surgical practice. Further research is necessary to advance the application of such integrative methods for the study and training of surgical teams, with the goal of enhancing team performance and patient safety.

## Supporting information

S1 Table Dataset (aggregated). (CSV)

S2 File Model output with effect sizes. (DOCX)
