# Supplementary material for: Dynamic patterns of communication and workload during cardiac surgery: An explorative study
Source: PLoS One. 2026 Jun 30;21(6):e0352703. doi: 10.1371/journal.pone.0352703 (PMC13318023; doi:10.1371/journal.pone.0352703)
Supplement: S2 File — (DOCX) [file pone.0352703.s002.docx]

**S2. Supplemental Material: Effect Sizes of Model Outcomes**

1. Case-Relevant Communication by Phase

Effect sizes (Rate Ratios):

| *Predictor* | *Rate Ratio* | *95% CI* | *p-value* |
| --- | --- | --- | --- |
| Intercept | 4.269 | 3.977–4.582 | 0.000 |
| phase: 1 | 0.801 | 0.751–0.855 | 0.000 |
| phase: 2 | 1.542 | 1.445–1.646 | 0.000 |
| phase: 3 | 0.768 | 0.723–0.816 | 0.000 |
| phase: 4 | 1.389 | 1.303–1.481 | 0.000 |

Overall Model Fit (R^2^)

| *Metric* | *Value* |
| --- | --- |
| Marginal R² (fixed effects) | 0.643 |
| Conditional R² (fixed + random) | 0.645 |

1. Case-Irrelevant Communication by Phase

Effect Sizes (Rate Ratios)

| *Predictor* | *Rate.Ratio* | *95% CI* | *p.value* |
| --- | --- | --- | --- |
| Intercept | 0.565 | 0.478–0.668 | 0.000 |
| phase: 1 | 0.887 | 0.762–1.033 | 0.124 |
| phase: 2 | 0.890 | 0.752–1.053 | 0.173 |
| phase: 3 | 0.987 | 0.856–1.137 | 0.853 |
| phase: 4 | 0.856 | 0.728–1.006 | 0.059 |

Model Fit (R2):

| *Metric* | *Value* |
| --- | --- |
| Marginal R² (fixed effects) | 0.128 |
| Conditional R² (fixed + random) | 0.133 |

1. Maximum Workload by Phase and Role

Effects Sizes, Partial Eta-squared (η²)

| *Predictor* | *Partial* *η²* |
| --- | --- |
| phase | 0.155 |
| role | 0.012 |
| phase × role | 0.146 |

Model Fit (R2)

| *Metric* | *Value* |
| --- | --- |
| Marginal R² (fixed effects) | 0.246 |
| Conditional R² (fixed + random) | 0.286 |

1. Mean Workload by Phase and Role

Effects Sizes, Partial Eta-squared (η²)

| *Predictor* | *Partial* *η²* |
| --- | --- |
| phase | 0.134 |
| role | 0.013 |
| phase × role | 0.152 |

| *Metric* | *Value* |
| --- | --- |
| Marginal R² (fixed effects) | 0.240 |
| Conditional R² (fixed + random) | 0.275 |

1. CRC by workload, phase & role

Effect sizes (Rate Ratios):

| *Predictor* | *Rate.Ratio* | *95% CI* | *p.value* |
| --- | --- | --- | --- |
| Intercept | 6.102 | 5.498–6.773 | 0.000 |
| hrv × | 1.064 | 0.926–1.221 | 0.382 |
| phase × 1 | 0.734 | 0.599–0.9 | 0.003 |
| phase × 2 | 1.149 | 0.961–1.374 | 0.127 |
| phase × 3 | 0.785 | 0.683–0.902 | 0.001 |
| phase × 4 | 1.959 | 1.709–2.246 | 0.000 |
| role × 1 | 0.678 | 0.598–0.769 | 0.000 |
| role × 2 | 0.712 | 0.627–0.808 | 0.000 |
| hrv × :phase1 | 1.146 | 0.847–1.551 | 0.378 |
| hrv × :phase2 | 1.465 | 1.103–1.944 | 0.008 |
| hrv × :phase3 | 0.930 | 0.731–1.185 | 0.558 |
| hrv × :phase4 | 0.692 | 0.535–0.895 | 0.005 |
| hrv × :role1 | 1.278 | 1.04–1.572 | 0.020 |
| hrv × :role2 | 0.778 | 0.627–0.965 | 0.022 |
| phase × 1:role1 | 1.306 | 0.988–1.727 | 0.061 |
| phase × 2:role1 | 0.766 | 0.583–1.007 | 0.056 |
| phase × 3:role1 | 0.917 | 0.741–1.137 | 0.431 |
| phase × 4:role1 | 1.374 | 1.125–1.679 | 0.002 |
| phase × 1:role2 | 0.785 | 0.566–1.091 | 0.149 |
| phase × 2:role2 | 1.333 | 1.036–1.716 | 0.026 |
| phase × 3:role2 | 0.843 | 0.698–1.018 | 0.075 |
| phase × 4:role2 | 1.016 | 0.839–1.229 | 0.873 |
| hrv × :phase1:role1 | 0.782 | 0.512–1.196 | 0.257 |
| hrv × :phase2:role1 | 1.103 | 0.705–1.725 | 0.667 |
| hrv × :phase3:role1 | 0.582 | 0.394–0.859 | 0.006 |
| hrv × :phase4:role1 | 0.955 | 0.671–1.359 | 0.798 |
| hrv × :phase1:role2 | 1.158 | 0.706–1.899 | 0.561 |
| hrv × :phase2:role2 | 0.984 | 0.646–1.5 | 0.942 |
| hrv × :phase3:role2 | 2.007 | 1.414–2.848 | 0.000 |
| hrv × :phase4:role2 | 0.879 | 0.579–1.335 | 0.545 |

Model Fit (R2):

| *Metric* | *Value* |
| --- | --- |
| Marginal R² (fixed effects) | 0.703 |
| Conditional R² (fixed + random) | 0.758 |

1. CIC by workload, phase & role

| Effect sizes (Rate Ratios): | | | |
| --- | --- | --- | --- |
| *Predictor* | *Rate.Ratio* | *95% CI* | *p.value* |
| Intercept | 1.338 | 0.97–1.846 | 0.076 |
| hrv × | 0.387 | 0.276–0.543 | 0.000 |
| phase × 1 | 1.570 | 1.053–2.341 | 0.027 |
| phase × 2 | 1.036 | 0.636–1.689 | 0.887 |
| phase × 3 | 1.059 | 0.795–1.411 | 0.696 |
| phase × 4 | 0.659 | 0.449–0.968 | 0.034 |
| role × 1 | 1.761 | 1.365–2.27 | 0.000 |
| role × 2 | 0.536 | 0.4–0.717 | 0.000 |
| hrv × :phase1 | 0.589 | 0.309–1.122 | 0.108 |
| hrv × :phase2 | 0.630 | 0.278–1.427 | 0.268 |
| hrv × :phase3 | 0.966 | 0.579–1.613 | 0.895 |
| hrv × :phase4 | 1.208 | 0.551–2.647 | 0.637 |
| hrv × :role1 | 0.930 | 0.593–1.457 | 0.751 |
| hrv × :role2 | 0.955 | 0.554–1.647 | 0.869 |
| phase × 1:role1 | 0.780 | 0.465–1.309 | 0.347 |
| phase × 2:role1 | 1.895 | 1.032–3.481 | 0.039 |
| phase × 3:role1 | 0.809 | 0.558–1.173 | 0.264 |
| phase × 4:role1 | 1.010 | 0.595–1.717 | 0.970 |
| phase × 1:role2 | 1.621 | 0.859–3.058 | 0.136 |
| phase × 2:role2 | 1.647 | 0.822–3.298 | 0.159 |
| phase × 3:role2 | 0.884 | 0.591–1.321 | 0.546 |
| phase × 4:role2 | 0.553 | 0.314–0.974 | 0.040 |
| hrv × :phase1:role1 | 1.177 | 0.505–2.748 | 0.706 |
| hrv × :phase2:role1 | 0.507 | 0.179–1.439 | 0.202 |
| hrv × :phase3:role1 | 1.441 | 0.715–2.905 | 0.307 |
| hrv × :phase4:role1 | 0.884 | 0.317–2.47 | 0.815 |
| hrv × :phase1:role2 | 0.537 | 0.186–1.552 | 0.251 |
| hrv × :phase2:role2 | 0.651 | 0.192–2.21 | 0.491 |
| hrv × :phase3:role2 | 1.207 | 0.546–2.669 | 0.642 |
| hrv × :phase4:role2 | 1.860 | 0.495–6.985 | 0.358 |

| Model Fit (R2): | |
| --- | --- |
| *Metric* | *Value* |
| Marginal R² (fixed effects) | 0.224 |
| Conditional R² (fixed + random) | 0.506 |

1. Others’ CRC by workload phase & role

| Effect sizes (Rate Ratios): | | | |
| --- | --- | --- | --- |
| *Predictor* | *Rate.Ratio* | *95% CI* | *p.value* |
| Intercept | 11.806 | 10.773–12.938 | 0.000 |
| hrv × | 0.974 | 0.887–1.069 | 0.578 |
| phase × 1 | 0.957 | 0.844–1.085 | 0.488 |
| phase × 2 | 0.968 | 0.838–1.118 | 0.659 |
| phase × 3 | 0.896 | 0.812–0.989 | 0.029 |
| phase × 4 | 1.099 | 0.98–1.233 | 0.108 |
| role × 1 | 1.304 | 1.206–1.41 | 0.000 |
| role × 2 | 1.438 | 1.338–1.545 | 0.000 |
| hrv × :phase1 | 1.056 | 0.876–1.273 | 0.567 |
| hrv × :phase2 | 1.389 | 1.115–1.73 | 0.003 |
| hrv × :phase3 | 0.886 | 0.76–1.033 | 0.121 |
| hrv × :phase4 | 0.944 | 0.777–1.148 | 0.566 |
| hrv × :role1 | 0.957 | 0.839–1.091 | 0.510 |
| hrv × :role2 | 1.000 | 0.882–1.133 | 0.994 |
| phase × 1:role1 | 0.919 | 0.781–1.082 | 0.312 |
| phase × 2:role1 | 1.417 | 1.183–1.697 | 0.000 |
| phase × 3:role1 | 1.120 | 0.994–1.261 | 0.062 |
| phase × 4:role1 | 0.922 | 0.791–1.075 | 0.300 |
| phase × 1:role2 | 1.141 | 0.973–1.338 | 0.104 |
| phase × 2:role2 | 0.893 | 0.75–1.064 | 0.207 |
| phase × 3:role2 | 0.834 | 0.749–0.929 | 0.001 |
| phase × 4:role2 | 1.034 | 0.911–1.174 | 0.605 |
| hrv × :phase1:role1 | 0.989 | 0.764–1.281 | 0.935 |
| hrv × :phase2:role1 | 0.686 | 0.505–0.933 | 0.016 |
| hrv × :phase3:role1 | 1.160 | 0.937–1.435 | 0.173 |
| hrv × :phase4:role1 | 1.014 | 0.778–1.32 | 0.921 |
| hrv × :phase1:role2 | 0.874 | 0.68–1.125 | 0.296 |
| hrv × :phase2:role2 | 1.126 | 0.841–1.509 | 0.426 |
| hrv × :phase3:role2 | 1.354 | 1.11–1.651 | 0.003 |
| hrv × :phase4:role2 | 0.873 | 0.673–1.132 | 0.305 |

| Model Fit (R2): | |
| --- | --- |
| *Metric* | *Value* |
| Marginal R² (fixed effects) | 0.613 |
| Conditional R² (fixed + random) | 0.728 |

1. Others’ CIC by workload phase & role

| Effect sizes (Rate Ratios): | | | |
| --- | --- | --- | --- |
| *Predictor* | *Rate.Ratio* | *95% CI* | *p.value* |
| Intercept | 1.517 | 1.217–1.891 | 0.000 |
| hrv × | 1.353 | 1.062–1.725 | 0.015 |
| phase × 1 | 0.914 | 0.656–1.272 | 0.592 |
| phase × 2 | 0.686 | 0.459–1.027 | 0.067 |
| phase × 3 | 1.502 | 1.171–1.927 | 0.001 |
| phase × 4 | 0.756 | 0.544–1.05 | 0.095 |
| role × 1 | 0.737 | 0.587–0.927 | 0.009 |
| role × 2 | 1.461 | 1.206–1.771 | 0.000 |
| hrv × :phase1 | 1.063 | 0.665–1.699 | 0.800 |
| hrv × :phase2 | 1.203 | 0.654–2.214 | 0.552 |
| hrv × :phase3 | 0.640 | 0.444–0.923 | 0.017 |
| hrv × :phase4 | 1.117 | 0.651–1.917 | 0.688 |
| hrv × :role1 | 1.375 | 0.94–2.011 | 0.100 |
| hrv × :role2 | 0.783 | 0.562–1.089 | 0.146 |
| phase × 1:role1 | 0.920 | 0.577–1.469 | 0.728 |
| phase × 2:role1 | 0.501 | 0.274–0.915 | 0.024 |
| phase × 3:role1 | 1.044 | 0.758–1.437 | 0.794 |
| phase × 4:role1 | 1.217 | 0.765–1.937 | 0.408 |
| phase × 1:role2 | 1.319 | 0.878–1.981 | 0.182 |
| phase × 2:role2 | 1.201 | 0.723–1.995 | 0.480 |
| phase × 3:role2 | 0.840 | 0.645–1.092 | 0.192 |
| phase × 4:role2 | 0.922 | 0.644–1.321 | 0.659 |
| hrv × :phase1:role1 | 1.134 | 0.549–2.343 | 0.733 |
| hrv × :phase2:role1 | 2.103 | 0.778–5.679 | 0.143 |
| hrv × :phase3:role1 | 1.178 | 0.669–2.075 | 0.569 |
| hrv × :phase4:role1 | 0.785 | 0.352–1.752 | 0.555 |
| hrv × :phase1:role2 | 0.637 | 0.335–1.211 | 0.169 |
| hrv × :phase2:role2 | 0.663 | 0.289–1.522 | 0.333 |
| hrv × :phase3:role2 | 1.135 | 0.698–1.846 | 0.610 |
| hrv × :phase4:role2 | 1.629 | 0.788–3.368 | 0.188 |

| Model Fit (R2): | |
| --- | --- |
| *Metric* | *Value* |
| Marginal R² (fixed effects) | 0.120 |
| Conditional R² (fixed + random) | 0.426 |

1. Phase Length by CIC & Phase

| Effects Sizes, Partial Eta-squared (η²) | |
| --- | --- |
| *Term* | *Partial η²* |
| CIC frequency per min | 0.018 |
| phase | 0.377 |
| CIC frequency × phase | 0.035 |

| Model Fit (R2): | |
| --- | --- |
| *Metric* | *Value* |
| Marginal R² (fixed effects) | 0.619 |
| Conditional R² (fixed + random) | 0.635 |

1. Phase Length by CRC & Phase

| Effects Sizes, Partial Eta-squared (η²) | |
| --- | --- |
| *Term* | *Partial η²* |
| CRC_frequencypermin | 0.089 |
| phase | 0.061 |
| CRC_frequencypermin:phase | 0.045 |

| Model Fit (R2): | |
| --- | --- |
| *Metric* | *Value* |
| Marginal R² (fixed effects) | 0.677 |
| Conditional R² (fixed + random) | 0.703 |

1. Phase Length by Workload, Role & Phase

| Effects Sizes, Partial Eta-squared (η²) | |
| --- | --- |
| *Term* | *Partial* η² |
| max HRV | 0.086 |
| role | 0.009 |
| phase | 0.157 |
| max HRV × role | 0.007 |
| max HRV × phase | 0.021 |

| Model Fit (R2): | |
| --- | --- |
| *Metric* | *Value* |
| Marginal R² (fixed effects) | 0.645 |
| Conditional R² (fixed + random) | 0.726 |
